# Wasserstein Distance-Based Deep Leakage from Gradients

**DOI:** 10.3390/e25050810

**Published:** 2023-05-17

**Authors:** Zifan Wang, Changgen Peng, Xing He, Weijie Tan

**Affiliations:** 1 State Key Laboratory of Public Big Data, College of Computer Science and Technology, Guizhou University, Guiyang 550025, China; gs.zfwang20@gzu.edu.cn (Z.W.);; 2 Guizhou Big Data Academy, Guizhou University, Guiyang 550025, China; 3 Academic Affairs Office of Guizhou University for Nationalities, Guizhou Minzu University, Guiyang 550025, China; 4 Key Laboratory of Advanced Manufacturing Technology, Ministry of Education, Guizhou University, Guiyang 550025, China

**Keywords:** Wasserstein distance, gradient, inversion, image reconstruction

## Abstract

Federated learning protects the privacy information in the data set by sharing the average gradient. However, “Deep Leakage from Gradient” (DLG) algorithm as a gradient-based feature reconstruction attack can recover privacy training data using gradients shared in federated learning, resulting in private information leakage. However, the algorithm has the disadvantages of slow model convergence and poor inverse generated images accuracy. To address these issues, a Wasserstein distance-based DLG method is proposed, named WDLG. The WDLG method uses Wasserstein distance as the training loss function achieved to improve the inverse image quality and the model convergence. The hard-to-calculate Wasserstein distance is converted to be calculated iteratively using the Lipschit condition and Kantorovich–Rubinstein duality. Theoretical analysis proves the differentiability and continuity of Wasserstein distance. Finally, experiment results show that the WDLG algorithm is superior to DLG in training speed and inversion image quality. At the same time, we prove through the experiments that differential privacy can be used for disturbance protection, which provides some ideas for the development of a deep learning framework to protect privacy.

## 1. Introduction

With the enhancement of the performance of computer hardware, artificial intelligence fields such as machine learning and deep learning have ushered in new breakthroughs in massive data sample training. Distributed training reduces training time in large-scale data set training while improving the security of private data. In 2016, Google proposed the concept of [1,2] federated learning, which only transmits gradients during training [3,4,5], permitting multiple clients to train models together under their own private training data, so that model training can be carried out through shared gradients under the condition that the data are not available from the local server. This method is widely used in machine learning model training under the condition that private information is included in training data sets [6,7]. Because the model is updated by aggregating the gradient mean from multiple data points, the original private training data are considered impossible to recover. However, Zhu et al. [8] first proposed a gradient inversion method based on iterative optimization, which poses a threat to the security of platforms based on shared gradient cooperative training models such as federated learning.

**Figure 1:**
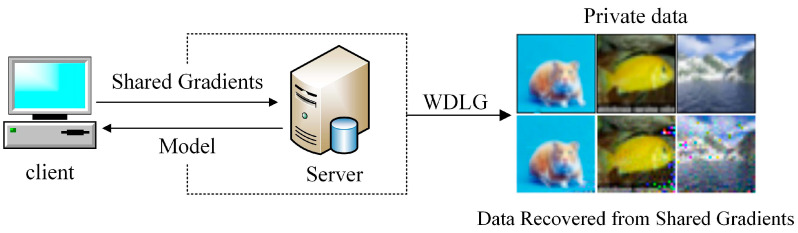
Inversion of original private training data via shared gradients.

In order to infer the information of training data from the gradient, there are early attempts at reversing the gradient to pursue the proxy information of the original data. The sample attributes of some training samples or data sets makes the gradient lead to the leakage of some shallow information. For example, the training binary classification model can determine whether the data record with certain attributes is included in the batch of other participants. However, this only causes a small amount of shallow information leakage. In “Deep Leakage from Gradient” [8], it was first found that the gradient carries private information about the importance of private training data. Its structure is shown in Figure 1. It minimizes the difference between the virtual gradient generated by the virtual data and the real gradient and then iteratively updates the noise data and the corresponding label. It only uses the simple Euclidean distance as the cost function between the virtual gradient and the real gradient, does not consider the directionality and geometric characteristics of the gradient distribution as the training data, and needs to be improved in generating high-quality inversion data.

In this paper, we propose the Wasserstein Deep Leakage from Gradient (WDLG) method. The proposed method uses Wasserstein distance [9] as loss function to fit virtual gradient and real gradient, which can provide more smooth results for the parameter update of the gradient descent method after derivation. Experiments are carried on MNIST, FashionMNIST, SVHN, and CIRFA10 datasets to verify the effectiveness of the DLG method. The major contributions of this paper are summarized threefold:

1. This paper proposes a gradient inversion attack algorithm based on DLG, which uses the Wasserstein distance to measure the distance between the virtual gradient and the real gradient.
2. Theoretical analysis is given about continuity and differentiability of Wasserstein distance; the analysis results show that Wasserstein distance substitution for Euclidean as a loss function of gradient is feasible in gradient inversion.
3. Experiments are carried on image data in public data set, and the result verifies that WDLG algorithm can invert images with better performance.

The rest of the paper is organized as follows: Section 2 describes the related work of federated learning, Wasserstein distance and gradient inversion, and the main significance of this paper. The Section 3 describes the theoretical derivation and improvement of the WDLG algorithm. Experimental verification is carried out in Section 4 to describe the experimental results and the advantages of the algorithm over the DLG algorithm. Conclusions and future work are presented in Section 5.

## 2. Related Work

Training data in distributed platforms such as federated learning can reduce training time and improve the security of private data, so they are widely used in machine learning model training, but their security is still threatened. Initially, some information was leaked through attribute inference, and until the emergence of DLG, the original information was completely leaked by iteratively optimizing the gradient. This makes the security of the training mechanism of federal learning shared gradient greatly threatened. More importantly, later researchers conducted more in-depth research based on DLG, from single image restoration, shallow network, low-resolution images to multi-image restoration, large-scale deep network, high-resolution images. Because the Wasserstein method is continuous and differentiable, it can provide a stable and smooth gradient when used as a training loss function. Therefore, the Wasserstein algorithm is used to optimize the gradient inversion algorithm to make its inversion ability stronger. This section conducts specific related work research from three directions: distributed training, Wasserstein algorithm, and gradient inversion.

### 2.1. Distributed Training

Large-scale machine learning model training requires a large amount of intensive computing. Many studies focus on distributed training to reduce machine learning model training time and ensure data privacy. The distributed training can be roughly divided into two types: centralized training [10,11] and decentralized training [12,13]. In centralized training, the gradient is aggregated first and then shared. The decentralized training exchange adjacent gradients. Either way, gradients are calculated to update local weights. The efficiency of distributed learning makes distributed learning achieve research breakthroughs at the algorithm level [14,15] and the framework level [16,17,18]. Most of them use stochastic gradient descent with strong stability as a training optimization algorithm.

In many practical applications, training data privacy needs to be protected. Joint learning has become a common strategy for training neural networks without transmitting data [19,20]. Model updates are exchanged between participants through gradients that are used to update the client private model locally on each participant. Therefore, multiple participants jointly train high-quality models only by sharing gradients so that private training data privacy is effectively protected.

The core idea is to share the gradient $\nabla L_{\theta }\left(x_{i},y_{i}\right)$ produced by back-propagation with the loss function $L_{\theta }\left(x_{i},y_{i}\right)$ in machine learning training consisting of minimized input image data $x_{i}$ and corresponding label group $y_{i}$. Then, update parameters θ by gradient $\nabla L_{\theta }\left(x_{i},y_{i}\right)$. The client exchanges gradient information in the server for weight update:

(1) $$\theta^{k+1}=\theta^{k}-\lambda \sum_{i=1}^{n}\nabla_{\theta }L_{\theta^{k}}\left(x_{i},y_{i}\right)$$

Each user completes a batch of training locally and sends the updated parameters $\theta^{k+1}$ back to the server. The training of the private model is completed by sharing only the gradient mean.

However, through the inference of membership, references [21,22,23] inferred some information in the training data. The recent emergence of references [24,25] has proved related gradient inversion attack techniques, and these attacks can recover the training data from the gradient information exchanged in the federated learning method under certain conditions. Therefore, the privacy protection ability of distributed machine learning is threatened.

### 2.2. Wasserstein (Earth-Mover) Distance

The origin of Wasserstein distance is the optimal transportation problem [26]. Also known as the Earth-Mover (EM) distance, it is defined as follows:

(2) $$W\left({\mathbb{P}}_{r},{\mathbb{P}}_{g}\right)=\underset{\gamma \in \prod \left({\mathbb{P}}_{r},{\mathbb{P}}_{g}\right)}{\inf}{𝔼}_{\left(x,y\right)∼\gamma }\left[\left\|x-y\right\|\right]$$

where $\prod \left({\mathbb{P}}_{r},{\mathbb{P}}_{g}\right)$ represents the set of all joint distributions $\gamma \left(x,y\right)$ with edge distributions ${\mathbb{P}}_{r}$ and ${\mathbb{P}}_{g}$. Intuitively, $\gamma \left(x,y\right)$ represents how much ‘mass’ must be transmitted from x to y in order to convert the distribution ${\mathbb{P}}_{r}$ to the distribution ${\mathbb{P}}_{g}$. Therefore, the EM distance is the ‘cost’ of the optimal transportation plan.

In deep learning, least squares, KL divergence, and cross entropy are often used as loss functions. These traditional distances are compared by the probability density function of the corresponding points, but most of them ignore the geometric characteristics between the probability distributions. EM distance can well reflect the geometric characteristics between probability distributions. The EM distance can find the Wasserstein average that is more capable of describing morphological features than the Euclidean average. It not only calculates the distance between the two distributions but also shows the evolution state matrix between the two distributions. Using EM distance as a deep learning loss function to measure the distance between two distributions can make the two distributions converge stably regardless of whether the distribution intersects. Based on the above advantages, more and more deep learning models use EM distance as the loss function. Among them, the most famous WGAN [27] uses EM distance to replace the loss function such as KL-divergence in the original GAN and obtains a better effect.

Gaiping [28] showed that if the parameter gradient is decomposed into its norm amplitude and direction, this amplitude only measures the local optimality of the data point relative to the current model. However, the high-dimensional direction of the gradient carries important information, so they propose to use an angle-based cost function. However, not only the gradient direction carries important information, but the geometric characteristics between the two gradient distributions also carry important information. EM distance reveals the geometric characteristics between the two distributions. Therefore, this paper uses EM distance to measure and optimize the distance between the virtual gradient and the original gradient.

### 2.3. Gradient Inversion

The earliest inversion of data using optimization methods was proposed by Wang et al. [24]. Zhu et al. [8] proposed the DLG method that completely relies on gradient difference minimization. By jointly optimizing the ‘pseudo’ label, the ‘pseudo’ noise data matches the real label and the real gradient to guide the iterative optimization of the noise data to obtain the original data. Zhao [29] proposed a single-hot label analysis method based on single-input reconstruction multi-class classification to extend the DLG method. This method recovers the original label before the iterative optimization training and no longer needs to train the matching label, so the image reconstruction speed and image accuracy are improved. In terms of inversion image quality assessment, Wei et al. [30] showed that a new image quality metric SSIM was proposed as an image similarity measure to guide the optimization of DLG. Gaiping et al. [28] used the peak signal-to-noise ratio as a quality measure while incorporating the prior of the image, which also opened up the study of adding regular terms to improve the accuracy of the image. After that, the references [31,32] completed the gradient inversion on high-fidelity, high-resolution data such as ImageNet and performed the next level task through the inverted image, such as continuous learning, knowledge transfer, etc. DeepInversion [33] produced good results on ImageNet image synthesis by batch normalization (BN) priors and feature distribution regularization. On this basis, reference [34] proposed a one-time batch label recovery algorithm, which proves that the gradient of the inverted batch can completely restore a single image with high fidelity and visual details of 224 pixel resolution.

It can be seen that optimized gradient inversion is based on DLG. DLG shows that it is possible to steal images from gradients by stealing images in pixels. In step t, each node i samples a small batch of samples $\left(x_{t,i},y_{t,i}\right)$ from its own data set to calculate the gradient $\nabla W_{t}$; the gradient is averaged on the server and used to update weights (Algorithm 1).
**Algorithm 1:** Deep Leakage from Gradients.**Input: **F (x; W): Differentiable machine learning model; W: parameter weights; ∇W: gradients calculated by training data.**Output:** private training data x, y1:procedure DLG (F, W, ∇W)
2:   $x^{\prime }\leftarrow N(0,1),{y^{\prime }}_{1}\leftarrow N(0,1)$
*Initialize dummy inputs and labels*.3:   **for**
i
← 1 to N **do**4:      $\nabla {W^{\prime }}_{i}\leftarrow \partial \ell (F({x^{\prime }}_{i},W_{t}),{y^{\prime }}_{i})/\partial W_{t}$
*Compute dummy gradients.*5:      ${𝔻}_{i}\leftarrow {\left\|\nabla {W^{\prime }}_{i}-\nabla W\right\|}^{2}$
***Second norm loss function.***6:      ${x^{\prime }}_{i+1}\leftarrow {x^{\prime }}_{i}-\eta \nabla_{{x^{\prime }}_{i}}{𝔻}_{i},{y^{\prime }}_{i+1}\leftarrow {y^{\prime }}_{i}-\eta \nabla_{{y^{\prime }}_{i}}{𝔻}_{i}$
*Update data to match gradients.*7:   **end for**

8:   **return**
${x^{\prime }}_{n+1},{y^{\prime }}_{n+1}$

9:**end procedure**


As shown in Algorithm 1, the DLG leaks training data through gradient matching, randomly initializing virtual input $x^{\prime }$ and label input $y^{\prime }$. Then, the virtual gradient $\nabla W^{\prime }$ is obtained by inputting the virtual data into the model. Minimize the distance between the virtual gradient $\nabla_{\theta }L_{\theta }\left(x^{\prime },y^{\prime }\right)$ and the original gradient $\nabla_{\theta }L_{\theta }\left(x,y\right)$ to restore the original input image x. The Euclidean distance of the loss function is shortened the gap between the virtual gradient and the real gradient. Therefore, the virtual data are similar to the real data by the virtual data update guided by the back propagation:

(3) $${x^{\prime }}^{*},{y^{\prime }}^{*}=\underset{x^{\prime },y^{\prime }}{\arg\min}{\left\|\nabla W^{\prime }-\nabla W\right\|}^{2}=\underset{x^{\prime },y^{\prime }}{\arg\min}{\left\|\frac{\partial \ell \left(F\left(x^{\prime },W\right),y^{\prime }\right)}{\partial W}-\nabla W\right\|}^{2}$$

The loss function is assumed to be second-order differentiable, which is optimized by gradient. We use the idea of DLG to iteratively minimize the difference between the virtual gradient and the original gradient and combine the Wasserstein distance as the loss function to improve the efficiency and accuracy of private data gradient inversion.

## 3. Method

In the original DLG, the training is unstable when the image is reconstructed by the Euclidean distance, and the image inversion effect is not good. In order to solve this problem, this paper uses a more stable algorithm in the training process, even when two distributions differ by a large margin and have no intersection at all, using the Wasserstein distance to measure the distance between them yields a stable, smooth gradient. This section combines the gradient inversion algorithm to iteratively optimize the gradient as a fitting object to obtain a gradient inversion loss function based on the Wasserstein distance. Theoretical analysis proves that the Wasserstein distance has continuity and differentiability, satisfying the basic conditions as a loss function. At the same time, the tag restoration algorithm proposed in the iDLG [29] algorithm is used to restore the tags in the original training data in advance, and the restored tags are used to guide the inversion algorithm to generate the training image during the iterative attack of gradient inversion so as to improve the inversion speed, efficiency, and image quality.

**Figure 2:**
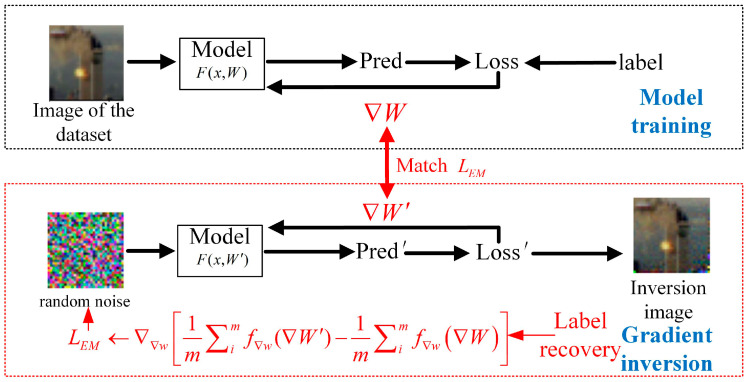
Overview of WDLG algorithm.

The core idea of the WDLG algorithm is shown Figure 2. When private training data are used to calculate and update the parameters, the WDLG algorithm gets dummy gradient from random noise data. The random noise is then guided into real private training data by minimizing the Wasserstein distance between true gradient ∇W and dummy $\nabla W^{\prime }$. Until the end of the iterative optimization, WDLG algorithm can obtain private training set information, resulting in privacy data leakage.

### 3.1. Wasserstein DLG (WDLG)

In Section 2, it is mentioned that the gradient inversion almost always uses the Euclidean loss function, but the Euclidean loss function cannot reflect the geometric characteristics between the parameters, especially in the case of optimization based on stochastic gradient descent, and the geometric characteristics between the two data distributions are closely related to the algorithm to optimize the trace trajectory. Therefore, the proposed method uses the EM distance to replace the Euclidean cost function in the DLG method. The EM cost function is as follows:

(4) $$𝔻\leftarrow \underset{\nu ∼\prod \left(p_{dummy},p_{true}\right)}{\inf}E_{(\nabla W^{\prime },\nabla W)∼\nu }\left[\left\|\nabla W^{\prime }-\nabla W\right\|\right]$$

where $\prod \left(p_{dummy},p_{true}\right)$ denotes all joint distributions of the distribution ${\mathbb{P}}_{dummy}$ of the virtual gradient and the distribution ${\mathbb{P}}_{true}$ of the real gradient. Each joint distribution $\nu \in \prod \left(p_{dummy},p_{true}\right)$ is used to characterize the cost of transforming ${\mathbb{P}}_{dummy}$ into ${\mathbb{P}}_{true}$ in continuous spatial distribution. Intuitively, ν indicates how much ‘mass’ must be moved from $\nabla W^{\prime }$ to ∇W to convert the ${\mathbb{P}}_{dummy}$ distribution into a ${\mathbb{P}}_{true}$ distribution. The EM distance is the optimal solution of this ‘quality’. $E_{(\nabla {W^{\prime }}_{i},\nabla W)∼\nu }\left[\left\|\nabla {W^{\prime }}_{i}-\nabla W\right\|\right]$ computes the expectation of the distance between the virtual gradient $\nabla W^{\prime }$ of the joint distribution ν and the real gradient ∇W; the optimal EM distance 𝔻 is obtained by infimum inf.

In this paper, the EM distance between the virtual gradient and the real gradient is calculated by the WDLG algorithm, and a more stable and efficient WDLG is proposed based on the EM distance gradient inversion method. As shown in Algorithm 2, the WDLG algorithm needs to randomly generate virtual data through the inverted machine learning model F(x;W), and the label $\left(x^{\prime },y^{\prime }\right)$ is introduced into the model F to train the virtual gradient $\nabla W^{\prime }$, calculating the EM distance 𝔻 between the quasi-gradient and the original gradient. However, using the WDLG algorithm must require the EM distance to be continuous and differentiable (proved in the latter part).

Since the minimized EM distance 𝔻$\left({\mathbb{P}}_{dummy},{\mathbb{P}}_{true}\right)$ is difficult to calculate, we use the method of Kantorovich–Rubinstein duality for expansion, so it is concluded that

(5) $$D\left({\mathbb{P}}_{dummy},{\mathbb{P}}_{true}\right)=\underset{{\left\|f\right\|}_{L}\leq 1}{\sup}{𝔼}_{\nabla w^{\prime }∼{\mathbb{P}}_{dummy}}[f(\nabla W^{\prime })]-{𝔼}_{\nabla w∼{\mathbb{P}}_{true}}[f(\nabla W)]$$

where the supremum is over all the 1-Lipschitz functions f: X → *R*. If ${\left\|f\right\|}_{L}\leq 1$ is replaced by ${\left\|f\right\|}_{L}\leq K$, the final result is $K\;\cdot \;D\left({\mathbb{P}}_{dummy},{\mathbb{P}}_{true}\right)$. If there is a parametrized series of functions ${\left\{f_{w}\right\}}_{w\in D}$, corresponding to K as K-Lipschitz, (5) can be transformed to (6).

(6) $$\underset{{\left\{f_{w}\right\}}_{w\in D}}{\max}{𝔼}_{\nabla w^{\prime }∼{\mathbb{P}}_{dummy}}\left[f\left(\nabla W^{\prime }\right)\right]-{𝔼}_{\nabla w∼{\mathbb{P}}_{true}}\left[f\left(\nabla W\right)\right]\;$$

If the supremum in (5) is attained for some w∈𝔻, this process would yield a calculation of 𝔻 up to a multiplicative constant. Furthermore, it can differentiate 𝔻 by back-propping through Equation (5) via estimating gradient.

Definition:

$$Q\left(\tilde{f},\varphi \right)={𝔼}_{\nabla w^{\prime }\sim dummy}[\tilde{f}(\nabla w^{\prime })]-{𝔼}_{\nabla w\sim true}[\tilde{f}(\nabla w)]$$

Since χ is compact, we know by the Kantorovich–Rubenstein duality [22] that there is an f∈F that attains the value

(7) $$𝔻\left({\mathbb{P}}_{dummy},{\mathbb{P}}_{true}\right)=\underset{\tilde{f}\in F}{\sup}Q(\tilde{f},\varphi )=Q(f,\varphi )$$

Definition $X^{*}(\varphi )=\left\{f\in F:Q(f,\varphi )=D\left({\mathbb{P}}_{dummy},{\mathbb{P}}_{true}\right)\right\}$. According to the above, $X^{*}(\varphi )$ is non-empty, so we have:

(8) $$\nabla_{\varphi }D\left({\mathbb{P}}_{dummy},{\mathbb{P}}_{true}\right)=\nabla_{\varphi }Q(f,\varphi ).\;=\nabla_{\varphi }[{𝔼}_{\nabla w^{\prime }\sim {\mathbb{P}}_{dummy}}f(\nabla w^{\prime })-{𝔼}_{\nabla w\sim {\mathbb{P}}_{true}}f(\nabla w)]$$

Therefore, adding batch m to the WDLG algorithm yields the algorithm iterative equation.

(9) $$\nabla D_{EM}{}_{i}=\nabla_{\nabla w}\left[\frac{1}{m}\sum_{i}^{m}f_{\nabla w}(\nabla W^{\prime })-\frac{1}{m}\sum_{i}^{m}f_{\nabla w}\left(\nabla W\right)\right]$$

Based on this training objective, we update the virtual data by gradient descent.

We randomly initialize the virtual data and the label $x^{\prime }\leftarrow N\left(0,1\right),y^{\prime }\leftarrow N\left(0,1\right)$ to calculate the virtual gradient $\nabla W^{\prime }$. The original private data are obtained by using Equation (9) as the loss function to calculate the loss value and optimally guide the virtual data by gradient descent. The Wasserstein Deep Leakage from Gradients process is given as follows.
**Algorithm 2:** Wasserstein Deep Leakage from Gradients.**Input: **F (x; W): differentiable machine learning model; W: model parameters;∇W: gradients calculated by training data; η$:\;\mathrm{learning}\;\mathrm{rate}.\;y^{*}$: tags recovered by tag recovery algorithm.**Output:** private training data x, y1:procedure WDLG (F, W, ∇W)
2:   $x^{\prime }\leftarrow N(0,1),y^{*}$
*Initialize dummy inputs and labels.*3:   **for**
i
← 1 to N **do**4:      $\nabla {W^{\prime }}_{i}\leftarrow \partial \ell (F(x_{i}{}^{\prime },W_{t}),y_{i}^{*})/\partial W_{t}$
*Compute dummy gradients.*5:      $\nabla D_{EM}{}_{i}\leftarrow \nabla_{\nabla w}\left[\frac{1}{m}\sum_{i=1}^{m}f_{\nabla w}(\nabla W^{\prime })-\frac{1}{m}\sum_{i=1}^{m}f_{\nabla w}\left(\nabla W\right)\right]$
***Wasserstein distance loss function.***6:      ${x^{\prime }}_{i+1}\leftarrow {x^{\prime }}_{i}-\eta \nabla_{{x^{\prime }}_{i}}L_{EM}{}_{i}$
*Update data to match gradients.*7:   **end for**
8:   **return**
${x^{\prime }}_{n+1},{y^{\prime }}_{n+1}$

9:**end procedure**


### 3.2. Continuity and Differentiability of EM Distance

Let ${\mathbb{P}}_{true}$ be the true distribution on χ; let ${\mathbb{P}}_{dummy}$ be the Gaussian variable on the space *Z*; and let the function g be χ denoting the EM distance function. The real distribution and the noise (dummy) distribution are denoted as $g_{true}\left(z\right)$ and $g_{dummy}\left(z\right)$. Define β and $\beta^{\prime }$ as two vectors in the real and dummy distributions, ${\mathbb{P}}_{dummy}$ as the random noise data distribution, and ${\mathbb{P}}_{true}$ as the gradient data distribution shared by the federal learning and other platforms; $\nu \in \prod \left({\mathbb{P}}_{\beta },{\mathbb{P}}_{\beta^{\prime }}\right)$, in the EM distance on the two distributions at random points noted as $\left(g_{\beta }\left(Z\right),g_{\beta^{\prime }}\left(Z\right)\right)$. According to the definition of Wasserstein distance,

(10) $$D\left(P_{\beta },P_{\beta^{\prime }}\right)\leq \int_{\chi \times \chi }\left\|x-y\right\|d\nu$$

$$\begin{array}{l} ={𝔼}_{(x,y)∼\nu }\left[\left\|x-y\right\|\right]\\ ={𝔼}_{z}\left[\left\|g_{\beta }(z)-g_{\beta^{\prime }}\left(z\right)\right\|\right] \end{array}$$

When g is continuous, there is $g_{\beta }(z)\to g_{\beta^{\prime }}\left(z\right)$, so $\left\|g_{\beta }-g_{\beta^{\prime }}\right\|\to 0$. Since χ converges, the distance between any two of these elements must be less than some constant M. $\left\|g_{\beta }\left(z\right)-g_{\beta^{\prime }}\left(z\right)\right\|\leq M$ The function is bounded and converges.

(11) $$D\left({\mathbb{P}}_{\beta },{\mathbb{P}}_{\beta^{\prime }}\right)\leq {𝔼}_{z}\left[\left\|g_{\beta }(z)-g_{\beta^{\prime }}\left(z\right)\right\|\right]\to_{\beta \to \beta^{\prime }}0$$

The Formula (12) is further obtained:

(12) $$\left|D\left({\mathbb{P}}_{true},{\mathbb{P}}_{\beta }\right)-D\left({\mathbb{P}}_{dummy},{\mathbb{P}}_{\beta^{\prime }}\right)\right|\leq D\left({\mathbb{P}}_{\beta },{\mathbb{P}}_{\beta^{\prime }}\right)\to_{\beta \to \beta^{\prime }}0\;$$

when the function g is continuous in the data distribution; at this time, $D\left({\mathbb{P}}_{dummy},{\mathbb{P}}_{true}\right)$ has continuity and satisfies the property of being a loss function. The function g satisfies the K-Lipschitz condition given a pair $\left(\beta ,x\right)$, a constant $L\left(\beta ,x\right)$, and an open set U such that $\left(\beta ,x\right)\in U$, $\left(\beta^{\prime },x^{\prime }\right)\in U$:

(13) $$\left\|g_{\beta }(x)-{g^{\prime }}_{\beta }(x^{\prime })\right\|\leq L\left(\beta ,x\right)(\left\|\beta -\beta^{\prime }\right\|+\left\|x-x^{\prime }\right\|)$$

Taking the expectation as well as the condition $x^{\prime }=x$,

(14) $${𝔼}_{x}\left[\left\|g_{\delta }(x)-g_{\delta^{\prime }}(x)\right\|\right]\leq \left\|\beta -\beta^{\prime }\right\|{𝔼}_{x}\left[L\left(\delta ,x\right)\right]$$

When $\left(\beta^{\prime },x^{\prime }\right)\in U$, one can define $U_{\beta }=\left\{\beta^{\prime }|(\beta^{\prime },x)\in U\right\}$. It is obvious that U as well as $U_{\beta }$ are open sets. $L(\beta )={𝔼}_{x}\left[L\left(\beta ,x\right)\right]$ can be derived by the continuity proved before

(15) $$\left|D({\mathbb{P}}_{dummy},{\mathbb{P}}_{\beta^{\prime }})-D({\mathbb{P}}_{true},{\mathbb{P}}_{\beta })\right|\leq D\left({\mathbb{P}}_{\beta },{\mathbb{P}}_{\beta^{\prime }}\right)\leq L\left(\beta \right)\left\|\beta -\beta^{\prime }\right\|$$

Above, $D\left({\mathbb{P}}_{dummy},{\mathbb{P}}_{true}\right)$ is continuous and differentiable under the K-Lipschitz condition.

EM distance is continuous and differentiable, conforms to the requirements of DLG loss, and can train inversion data to achieve the best state. In the following sections, we show the practical benefits of the proposed algorithm and provide the comparison with traditional DLG.

## 4. Experiment

In this paper, hardware and software environment are a Windows 10 operating system; the processor is Intel Core i5-9400F CPU@2.90GHz; and the memory is 16.00 GBGMet 64-bit operating system, using Python language and Pytorch framework to write experiments.

The learning rate is 0.1; the number of images generated is 300; the high-order gradient needs to be calculated; and 500 iterations are optimized for the image. The goal is to match gradients from all trainable parameters. The network used is LeNet network. The WDLG algorithm does not require model convergence or training completion, and its gradient inversion attack can occur at any time during the training process. All experiments use random initialization weights; virtual data and labels are random noises subject to Gaussian distribution. For more detailed information about specific tasks, see the following sections (Table 1).

### 4.1. Inversion Effect of WDLG on Image Classification

WDLG inversion is performed on the images of MNIST, Fashion MINIST, SVHN, and CIFAR-10 datasets. The fidelity of the generated image is measured by calculating the mean value of the inverted image and the original image, and the image comparison is performed in different batches of inversion.

**Figure 3:**
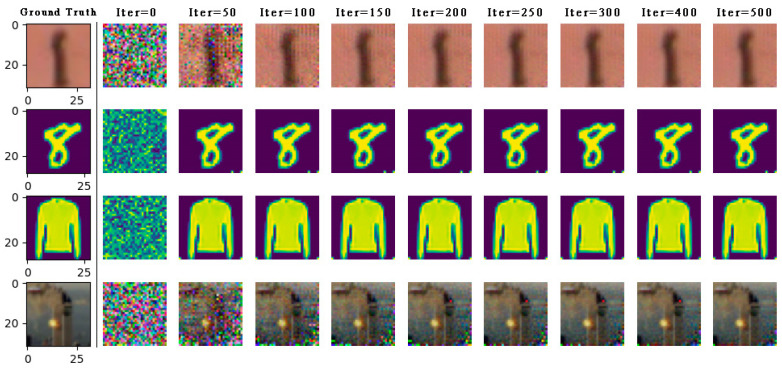
On the LeNet network model, the private data (part) of the four data sets are completely restored by WDLG algorithm in SVHN, MNIST, Fashion MNIST, and CIFAR-100.

As shown in Figure 3, the distance between gradients is minimized to guide the transformation of virtual data like real sample data. When the optimization is completed, the gradient inversion image is very similar to the real sample image, and there are almost no pseudo pixels that can be ignored. The WDLG algorithm fully recovers the images in these three datasets generate an image of 2828 pixels. Experiments show that monochrome images with clear background (MNIST, Fashion MNIST) are the easiest to recover, and SVHN, which is also handwritten but has a complex background, is slower than MNIST image inversion. The CIFAR-10 image with complex background and image needs more gradient inversion iterations to recover, and the fidelity of the inverted image is lower than that of the other three data sets.

**Figure 4:**
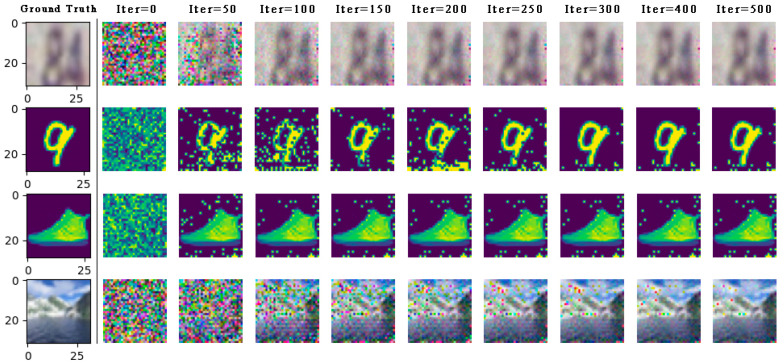
On the CNN6 network model, the private data (part) of the four data sets are completely restored by WDLG algorithm in SVHN, MNIST, Fashion MNIST, and CIFAR-100.

Next, as shown in Figure 4, We can still succeed in the gradient inversion under the CNN6 model with deeper depth, more parameters, and more complex network structure, but the convergence speed of the training image is slower than that of the LeNet network model.

**Figure 5:**
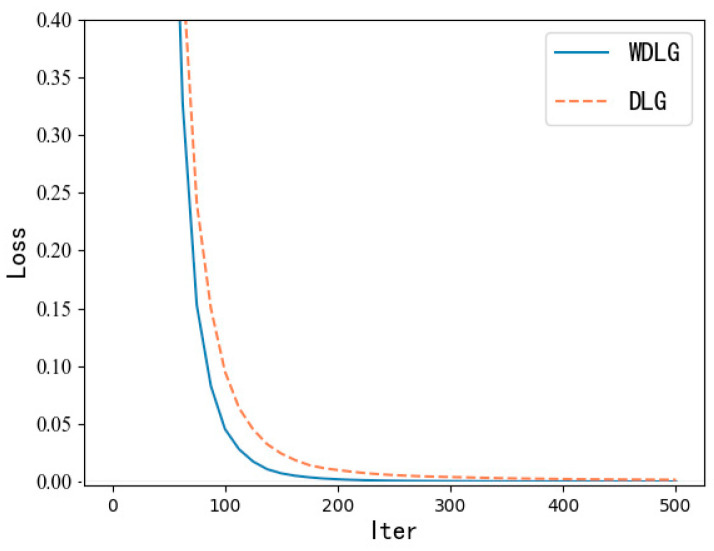
Training loss comparison.

As shown in Figure 5, in the CIFAR-10 dataset with the largest pixel value, when using the WDLG algorithm to train through 500 iterations, the training loss accuracy decreases faster than the DLG algorithm.

**Figure 6:**
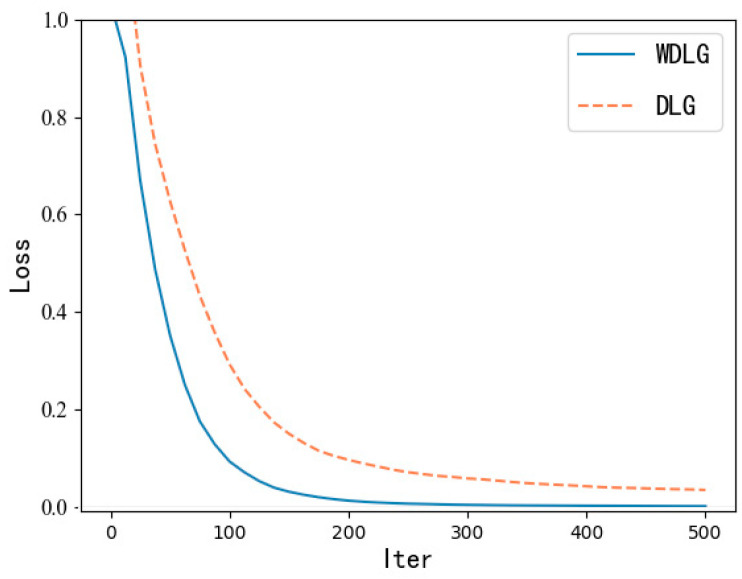
Fidelity comparison of reconstructed image.

In terms of image fidelity, as shown in Figure 6, we can observe that the mean square error of the image generated by the WDLG algorithm is lower than that of the DLG image—that is, WDLG generates an inverted image with higher fidelity. In summary, we intuitively show through experiments that the WDLG algorithm is superior to the DLG algorithm in terms of training loss accuracy and high-fidelity image inversion generation.

### 4.2. Calculation Comparison

**Figure 7:**
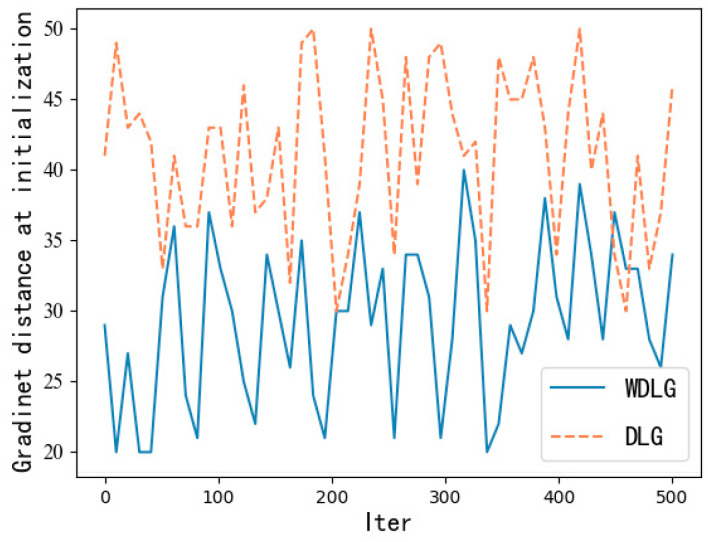
First loss calculation comparison.

In particular, there is an unexpected finding in training. Both WDLG and DLG algorithms randomly initialize the noise data subject to Gaussian distribution, but we find that when calculating the first gradient inversion iterative training loss distance, the WDLG algorithm always calculates a training loss that is less than twice that of the DLG algorithm. This means that the initial gradient of the WDLG distance is smaller than the original gradient distance, and the similarity is higher. Therefore, it has an advantage at the beginning of training iteration, as shown in Figure 7.

The attack success rates of the two schemes are compared under the FashionMNIST and CIFAR-10 data sets. The successful inversion image is divided by the total attack image, and the image inversion success rate shown in Table 2 is obtained.

The attack success rate of WDLG and DLG inversion algorithm is inversely proportional to the resolution of the inversion image. The higher the pixel of the image is, the more complex the image is, and the lower the success rate of inversion is. The success rate of gradient inversion attack of WDLG and DLG algorithm is almost the same, but WDLG is more dominant in running time.

When compared with the recursive gradient attack (RGAP) inversion method, RGAP provides a closed form recursive program to recover data from the gradient of the deep neural network. The mean square error is still used as the measure of image quality, and the experimental results are shown in the Table 3. Our method is almost consistent with the RGAP method in the inversion of image quality.

### 4.3. Experimental Results under Different Batches

**Figure 8:**
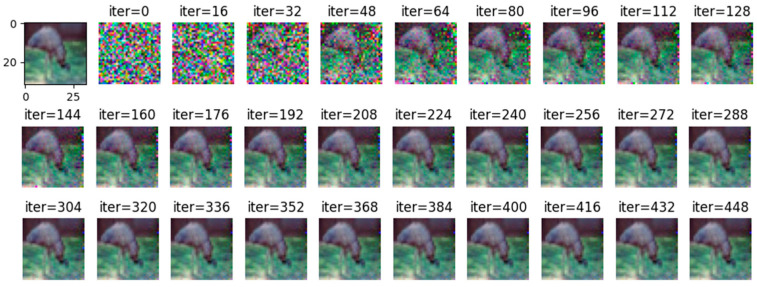
The image inversion recovery process results of WDLG trained 448 times in one batch of CIFAR-10 data set.

**Figure 9:**
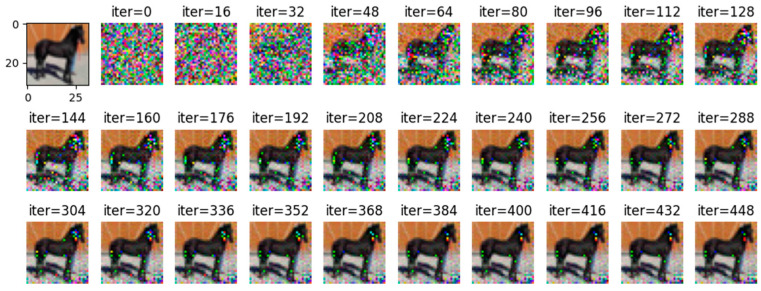
WDLG-trained 448 image inversion recovery process maps under 4 batches in CIFAR-10.

**Figure 10:**
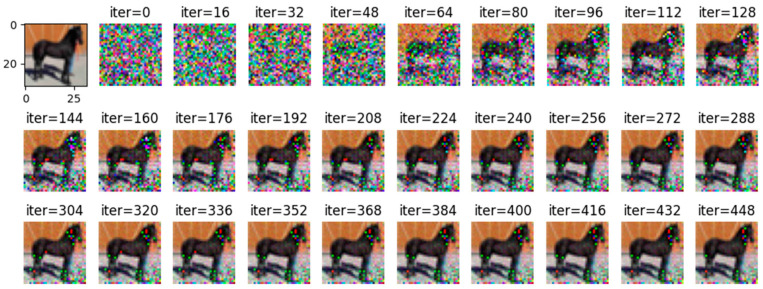
DLG-trained 448 image inversion recovery process maps under 4 batches in CIFAR-10.

We compared the training inversion attack on the image data in the CIAFR-10 dataset under batch 1 and batch 4 and found that the average total loss and mean square deviation between the real image and the inversion image of WDLG algorithm are smaller than those of DLG algorithm. Specific values are shown in the Table 4:

Comparing Figure 8 with Figure 9, it can be observed that all images are reconstructed under the WDLG method, and single batch image reconstruction quality is significantly higher than 4 batches image reconstruction. Comparing Figure 9 with Figure 10, it can be observed that the image quality reconstructed by WDLG algorithm is higher than that by DLG algorithm in 4 batches. However, the image inversion effect is worse than that of single batch WDLG. After 400 iterations of training, noisy data can still be observed, while single batch training can hardly see noise. However, compared with the same inversion batch DLG, the effect is better, and it can be obviously observed that the WDLG has less noise in the image inversion under the same training times during the whole training process.

### 4.4. Ablation Studies

In order to better enhance the ability of the algorithm and enrich our work, we use the label recovery algorithm proposed by iDLG on the basis of the original algorithm to recover the tags of the training data in advance and no longer invert the tags in the training process. Therefore, we also modify the whole algorithm design to make the gradient inversion algorithm have more accurate label for data recovery training, which can improve not only the inversion image quality and success rate but also the training convergence speed. Experiments are carried out on the original DLG algorithm under the condition of increasing label recovery and Wasserstein distance. A total of 500 iterative experiments are carried out under the LeNet network model and CIFAR10 dataset. When the training loss is lower than that, the image converges, and the attack is successful. The experiment is shown in Table 5:

Then, the training loss refers to the training error when the training process converges finally. Image quality refers to the mean square difference between the restored image and the original image. The smaller the difference is, the higher the image quality is. The convergence speed refers to the number of iterative training when the image is restored. The success rate of attack refers to the percentage of images that successfully leak image information for every 100 images of reverse attack.

It can be seen that when using the tag recovery algorithm to extract tags in advance, the inversion loss is reduced, and the image quality, the convergence speed, and the success rate of attack are improved. When using Wasserstein distance as loss function, compared with DLG inversion loss, image quality, convergence speed, and attack success rate are improved, but compared with label restoration algorithm, image quality and convergence speed do not change significantly.

### 4.5. Differential Privacy Disturbance Defense

For the Wasserstein gradient inversion algorithm proposed by us and a variety of previous gradient inversion algorithms, it is concluded that this kind of inversion attacks always match the virtual gradient with the real gradient through a set of virtual data input models, which makes the noise data iteratively updated and finally form the original data. Based on this, in the exploration of defense methods, adding noise to the gradient to make the gradient inversion attack worse is one of the most direct and effective defense methods. Therefore, we resist the gradient inversion attack algorithm by adding Gaussian differential privacy noise disturbance to the original gradient in the training model and carrying out simulation analysis (the experimental environment is consistent with the gradient inversion attack environment).

The experiment first adds differential privacy disturbance to the original gradient [35] and then uses Wasserstein gradient inversion algorithm to carry out gradient inversion attack.

**Figure 11:**
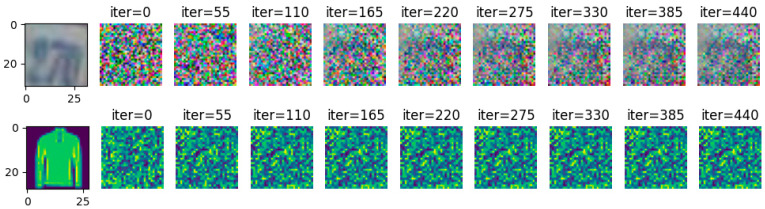
σ = 10, FashionMNIST, SVHN resisting gradient inversion effect.

Gradient inversion attacks are carried out on SVHN and FashionMNIST, respectively, in the case of noise of 10. The attack results are shown in Figure 11. In the case of 440 iterative attacks by the gradient inversion algorithm, the image information of the two data sets is effectively guaranteed and does not cause image information leakage.

**Figure 12:**
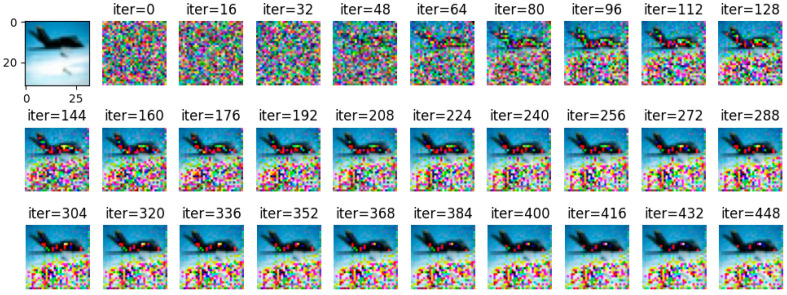
σ = 2, gradient inversion defense effect diagram under CIFAR-100.

By adjusting the noise to =2, =4, and =10, respectively, and using Wasserstein gradient inversion algorithm to attack the image in the CIFAR-100 data set, the image clearly shows that when the noise is =2, the training image under the iterative attack of 448 batches of gradient inversion does not completely disclose information but shows a certain degree of information leakage, as shown in Figure 12.

**Figure 13:**
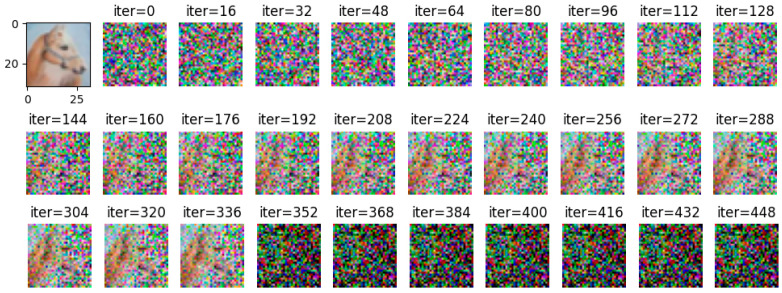
σ = 4, gradient inversion defense effect map under CIFAR-100.

When =4, the training image under the same iterative attack of 448 batches of gradient inversion can hardly see the information of the original image, but there is still a very small amount of data to show, as shown in Figure 13.

**Figure 14:**
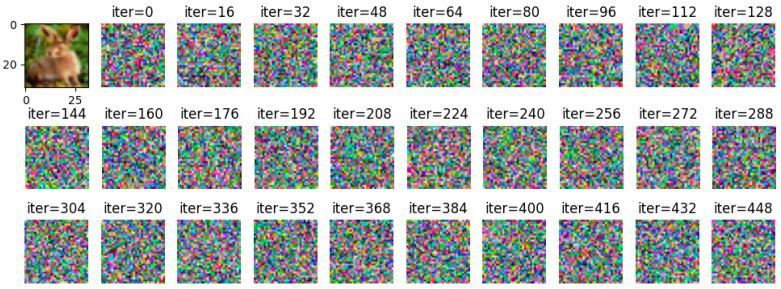
σ = 10, gradient inversion defense effect diagram under CIFAR-100.

When =10:00, the training image under the same iterative attack of 448 batches of gradient inversion cannot see the information of the original image at all, which completely defends the gradient inversion attack based on Wasserstein, as shown in Figure 14.

From the experimental results, we can see that when the noise increases gradually, the image quality of the Wasserstein gradient inversion attack decreases until no available information can be obtained, which realizes the defense of the gradient inversion attack algorithm. Therefore, the experimental results show that the defense method based on differential privacy disturbance in the original gradient is effective to resist the gradient inversion attack.

## 5. Conclusions

In this paper, a gradient inversion attack algorithm is proposed named WDLG, which applies Wasserstein distance-based Deep Leakage from Gradients to process image reconstruction for a given average gradient and can obtain higher quality inversion images in a shorter time. Theoretical derivation shows that EM distance is a continuous and differentiable function, which is fit for a loss function in depth learning gradient inversion attack series algorithms. We combine Kantorovich–Rubinstein duality and Lipschitz condition to calculate the WDLG algorithm by iterative calculation which solves the problem of Wasserstein distance being difficult to calculate. The experimental results show that the reconstructed image by WDLG algorithm in gradient inversion is almost the same as the original image; the image quality of the proposed method is better than DLG; and the reconstruction time is less.

We show that the training data will be leaked when the gradient of the deep learning network is shared on the dataset. At the same time, we prove through experiments that differential privacy can be used for disturbance protection, which provides some ideas for the development of a deep learning framework to protect privacy.

The image restored by WDLG inversion attack in high-resolution image is not good. The future work can be considered under the Wasserstein algorithm, by adding regular terms to constrain, in order to pursue higher quality image inversion effect.

## Figures and Tables

**Table 1:** Training parameter setting.

Training Parameter Setting	
Learning rate η	0.1
Number of iterations training N	500
Number of images generated	300
Inverted network model	LeNet
Random initialization weights	$x^{\prime }\leftarrow N(0,1),{y^{\prime }}_{1}\leftarrow N(0,1)$
Data set	MNIST, Fashion MINIST, SVHN, CIFAR-10, CIFAR-100

**Table 2:** Success rate of gradient inversion attacks under four datasets.

		Number of Iteration	Success Rate of Attack	Running Time
MNIST	DLG	448	0.82	1345
	WDLG	448	0.86	842
Fashion MNIST	DLG	448	0.84	1874
	WDLG	448	0.88	1026
SVHN	DLG	448	0.79	2115
	WDLG	448	0.86	1231
CIFAR-100	DLG	448	0.76	2315 s
	WDLG	448	0.81	1510 s

**Table 3:** Comparison of WDLG, DLG, and RGAP inversion algorithms.

	LeNet + MNIST	CNN6 + MNIST	LeNet + CIFAR10	CNN6 + CIFAR10
DLG	0.0037 ± 0.00082	0.015 ± 0.0053	0.013 ± 0.0012	0.0513 ± 0.034
RGAP	0.0012 ± 0.00054	0.0068 ± 0.0012	0.0048 ± 0.00081	0.0258 ± 0.016
WDLG	0.0014 ± 0.00069	0.0057 ± 0.0029	0.0045 ± 0.00075	0.028 ± 0.0064

**Table 4:** Comparison of data between WDLG and DLG in batch 1 and batch 4.

Learning Rate η	Batch Size	Loss	MSE
$D_{EM}\left(x^{\prime },y^{\prime }\right)$	Batch size 1	$4.48\times 10^{-5}$	$1.39\times 10^{-2}$
	Batch size 4	$1.13\times 10^{-4}$	$4.56\times 10^{-3}$
$D_{DLG}\left(x^{\prime },y^{\prime }\right)$	Batch size 1	$4.73\times 10^{-5}$	$4.34\times 10^{-3}$
	Batch size 4	$1.11\times 10^{-4}$	$7.93\times 10^{-3}$

**Table 5:** Ablation studies.

	Loss	Image Quality	Number of Iteration	Success Rate of Attack
DLG	$8.06\times 10^{-5}$	$1.3\times 10^{-2}$	300	0.74
+Label recovery algorithm	$6.4\times 10^{-5}↑$	$4.56\times 10^{-3}↑$	150 ↑	0.78 ↑
+Wasserstein Distance	$5.23\times 10^{-5}↑$	$4.6\times 10^{-3}↓$	140 ↑	0.80 ↑

↑/↓ indicates an increase/decrease compared to the previous line of data.

## Data Availability

The data used to support the finding of this study are included in the article.
